# The Use of Augmented Reality for Navigation in Minimally Invasive Abdominal and Thoracic Soft-Tissue Surgery: A Systematic Review

**DOI:** 10.3390/s26061962

**Published:** 2026-03-20

**Authors:** Inga Steinberga, Victor Gabriel El-Hajj, Laura Cercenelli, Mario Romero, Kenny A. Rodriguez-Wallberg, Erik Edström, Adrian Elmi-Terander

**Affiliations:** 1 Laboratory of Translational Fertility Preservation, Department of Oncology–Pathology, Karolinska Institute, 171 64 Stockholm, Sweden; 2 Department of Gynecology, Division of Gynecology and Reproduction, Karolinska University Hospital, 141 86 Stockholm, Sweden; 3 Department of Clinical Neuroscience, Karolinska Institute, 171 65 Stockholm, Sweden; victor.gabriel.elhajj@stud.ki.se (V.G.E.-H.); erik.edstrom.1@ki.se (E.E.); adrian.elmi.terander@ki.se (A.E.-T.); 4 Laboratory of Bioengineering–eDIMES Lab, Department of Medical and Surgical Sciences, University of Bologna, 40138 Bologna, Italy; laura.cercenelli@unibo.it; 5 Department of Science and Technology, Linköping University, 601 74 Linköping, Sweden; mario.romero@liu.se; 6 Department of Reproductive Medicine, Division of Gynecology and Reproduction, Karolinska University Hospital, 141 86 Stockholm, Sweden; 7 Capio Spine Center, 194 02 Stockholm, Sweden

**Keywords:** abdominal, augmented reality, minimally invasive surgery, soft-tissue surgery, systematic review, thoracic, computer-assisted surgery

## Abstract

**Highlights:**

**What are the main findings?**

- Evidence supporting the use of augmented reality (AR) technology in minimally invasive abdominal and thoracic soft-tissue surgery is mostly limited to small-scale or preliminary studies and lacks comprehensive validation. In gynecologic surgery, current research is scarce and does not provide adequately validated outcomes.
- Guidance with AR systems requires deformable anatomical tracking and multimodal intraoperative data fusion to ensure safety and effectiveness.

**What are the implications of the main findings?**

- AR systems offer clinical potential as guidance tools, but adoption is limited by small sample sizes, varied study designs, and non-standardized reporting. Larger, high-quality studies, standardized training, and objective evaluation metrics are needed.
- Successful AR-assisted surgery relies on real-time, deformable anatomical tracking with multimodal imaging. This enhances alignment, precision, and safety, especially when using robotic platforms.

**Abstract:**

Surgical navigation and augmented reality (AR) are widely used in neurosurgery, spinal surgery, and orthopedics. However, their use in minimally invasive abdominal and thoracic soft-tissue surgery is limited, as tracking deformable, mobile organs is challenging. Recent advances in AR may address these challenges to improve intraoperative navigation. This systematic review, registered in PROSPERO (2024) and based on PRISMA guidelines, analyzes literature from 2014 to 2024 about AR in minimally invasive abdominal and thoracic soft-tissue surgery. It identifies target organs, describes AR hardware and software, and evaluates accuracy levels, usability outcomes, clinical benefits, technical limitations, and research needs. Searches of PubMed, Web of Science, and Embase for English-language studies found 1297 records, of which only 28 (2%) met the inclusion criteria. Nearly half (*n* =12; 42%) focused on liver surgery; none on gynecologic surgery. The AR devices varied in tracking methods, image processing, visualization, and display. Overall, AR improved anatomical guidance and procedural planning, especially in complex surgeries. Integration with robotic systems may further boost visualization, precision, and workflow, though challenges remain in standardization, large-cohort validation, and workflow integration.

## 1. Introduction

Surgical navigation has been used for decades in neurosurgery, spine surgery [1], and orthopedic procedures [2], relying on fixed anatomical references for patient coregistration. More recently, augmented reality (AR) has emerged, adding digital information from imaging modalities such as MRI, CT, and 3D US directly to the surgeon’s view [3]. Fusion, the process of combining information from different sources within AR systems, can be achieved through separate video displays, projection techniques, or, most commonly, head-mounted displays (HMDs) [4]. However, according to this systematic review, only a few studies reported the use of HMDs in AR [5,6]. HMDs improve intraoperative orientation by overlaying anatomical structures onto the patient, enhance spatial understanding, and reduce external distraction, but their weight (approximately 400–800 g) may cause neck strain, motion sickness, and visual fatigue during prolonged use.

In contrast, surgical navigation in abdominal and thoracic procedures has progressed slowly. The main challenge is maintaining accurate coregistration. Aligning preoperative 3D models with intraoperative anatomy is technically demanding and tracking real-time deformation of mobile soft tissues—such as the liver, kidneys, lungs, and uterus—is difficult due to continuous changes during surgery. Real-time algorithms are needed to compensate for tissue elasticity and manipulation. AR offers promising solutions to enhance navigation in abdominal and pelvic surgery by overlaying key structures (e.g., blood vessels or tumors) onto the patient, improving orientation and safety during complex, minimally invasive procedures [3]. Displaying vital structures in real time, AR helps prevent accidental injuries and improves both preoperative and intraoperative planning. AR also enhances surgical training by providing a clear, stable combined view of real anatomy [7,8]. For example, robotic platforms with stabilized endoscopic cameras offer reliable visualization, allowing accurate AR overlays [9,10].

The establishment of AR in minimally invasive abdominal and thoracic soft-tissue surgery depends on several key technological strategies. First, computational segmentation and 3D model creation generate virtual anatomical models for projection onto the surgical field, with the accuracy of these overlays depending on the quality of image acquisition and segmentation. Second, registration aligns preoperative 3D models with the patient’s intraoperative anatomy. Because soft tissues change shape and position during surgery, reliable registration requires strong algorithms and dependable landmarks. Real-time deformation tracking is critical, as organ deformation is the main challenge in soft-tissue surgery compared to bone-based navigation [11,12,13,14]. Third, real-time motion compensation and continuous tracking keep AR overlays aligned, even when the endoscope moves. Manual manipulation and repositioning of the endoscope create challenges for computer-assisted systems, making it difficult to maintain dynamic, real-time adaptation of AR overlays [9,10]. Finally, AR systems must also synchronize with physiological movements, such as breathing and heartbeat, to reduce motion-induced errors.

**Figure 1:**
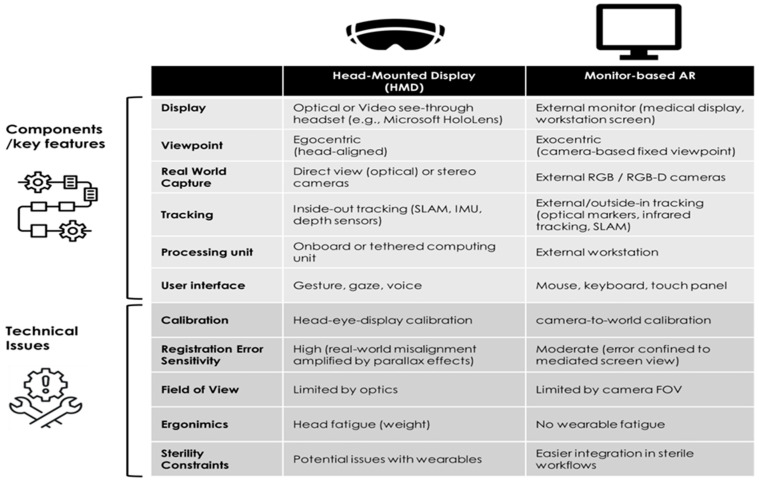
Simple Graphical Representation of VR Head-Mounted Display (HDM) System and Non-HMD (Monitor-Based) VR Setup.

Figure 1 describes differences between VR Head-Mounted Display (HMD) System and Non-HMD (Monitor-Based) VR.

This systematic review aims to capture the current state of the art in the use of augmented reality (AR) for navigation in minimally invasive abdominal and thoracic soft-tissue surgery. It explores and summarizes literature from 2014 to 2024, focusing on highly mobile anatomical structures that may change shape during procedures. Following PRISMA guidelines, we detail the methodology and present key findings from the included studies, covering general setup, devices employed, accuracy levels, usability outcomes, and challenges. The review concludes with the main findings and future perspectives.

Our specific goals are to investigate the following research questions:

- In which organs and procedures of minimally invasive abdominal and thoracic soft-tissue surgery has AR been used?
- How is AR applied to highly mobile anatomical structures whose shape changes during surgical procedures?
- What devices are used in recent research on this topic?
- What accuracy levels and usability outcomes have been reported for the augmentation?
- What challenges and unresolved problems still need to be addressed to advance the development and effective use of AR?

## 2. Materials and Methods

This systematic review was conducted following the Preferred Reporting Items for Systematic Reviews and Meta-Analyses (PRISMA) guidelines [15]. The PRISMA 2020 checklist and further methodological details are available in the Supporting Information [Files S1–S4].

The review protocol is registered in the International Prospective Register of Systematic Reviews (PROSPERO) [16] (Registration ID: CRD42024597021; registration date: 24 October 2024).

### 2.1. Eligibility Criteria

All original studies published between 2014 and 2024, written in English, and focusing on AR applications in minimally invasive abdominal and thoracic soft-tissue surgery on humans were eligible for inclusion in this systematic review (Table 1).

### 2.2. Types of Studies

The systematic review encompasses published, peer-reviewed original human studies, including randomized trials, observational studies, and case series with a sample size of 4 or more.

Non-original publications, including reviews, editorials, letters to the editor, conference abstracts, unpublished studies, and prior systematic reviews, were excluded. Additionally, studies on augmented reality navigation in orthopedic, neurosurgical, vascular, and plastic surgery were omitted.

Single case reports and studies with fewer than 4 participants were excluded due to their limited sample sizes, which reduces the level of evidence.

### 2.3. Type of Population

Studies that recruited adults aged 18–64 years were included and were considered representative of adult age.

Studies that recruited adolescents aged ≤17 years or adults aged ≥65 years were excluded as falling outside the adult age range.

### 2.4. Type of Intervention

Only articles focusing on the use of AR applications on humans within minimally invasive abdominal and thoracic soft-tissue surgery were considered for inclusion in this review. Studies addressing the use of other technologies of extended reality (XR) such as mixed reality (MR) and virtual reality (VR) were excluded.

### 2.5. Type of Comparators

There were no restrictions with respect to the type of comparator.

Data are categorized according to the following:

- Comparison is made between groups with and without AR,
- Technology is classified according to types of devices and the use of head-mounted displays (HMDs),
- The use of additional technologies in conjunction with AR is documented.

This categorization will form the basis for descriptive narrative synthesis.

### 2.6. Type of Outcome Measures

Data is pooled as total counts and frequencies to assess the state of the literature on the use of AR in abdominal and thoracic soft-tissue surgery.

The certainty of evidence is evaluated by examining the individual risk of bias in each study and the strength of the combined body of evidence.

No subgroup investigation is planned.

### 2.7. Databases and Search Strategy

Three electronic bibliographic databases relevant to the medical field—PubMed, Web of Science, and Embase—were utilized. The literature search was confined to the period 2014 to 2024 and was restricted to articles published in English. The database search was completed on 28 October 2024.

### 2.8. Study Selection

**Figure 2:**
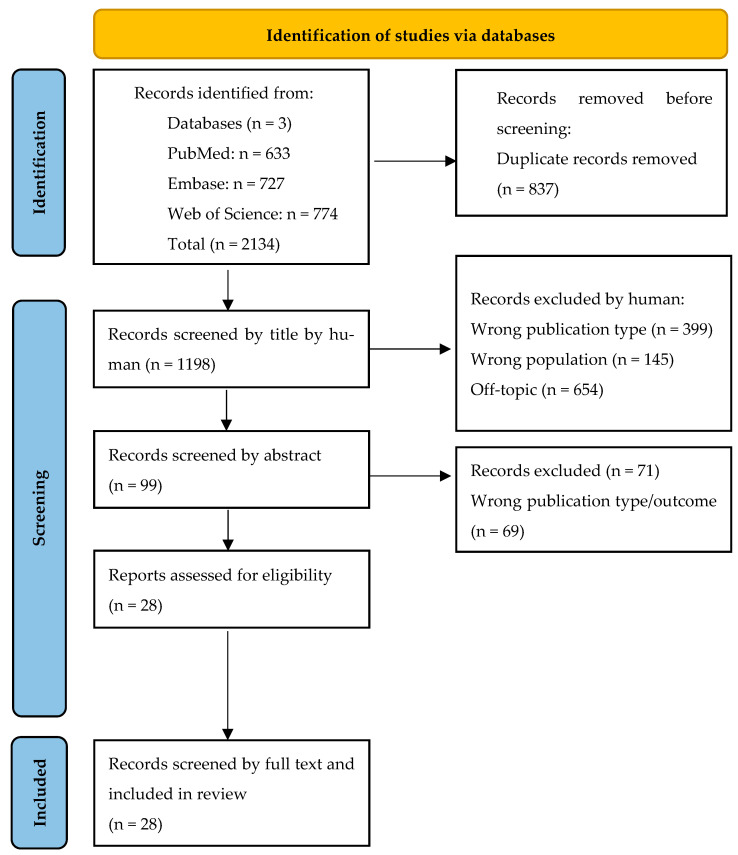
PRISMA 2020 Flow Diagram for Systematic Reviews [15].

The search identified 2134 papers across databases. After removing duplicates, 1297 papers remained. These records were uploaded to Rayyan [17]. Two independent, non-blinded reviewers (I.S. and V.G.E.-H.) screened 1297 records by title and then by abstract. Disagreements concerning two articles were resolved through discussion and consultation with a third reviewer (K.A.R-W.). Subsequently, data extraction from the full texts of the 28 remaining articles was conducted by two independent, non-blinded reviewers (I.S. and L.C.). Ultimately, 28 studies were deemed eligible for inclusion in this review, as illustrated in the PRISMA flow chart in Figure 2.

### 2.9. Data Extraction

Data from selected records were extracted using a predefined template. This template included general information such as:

- Study title, study purpose, first author, and record publication year, surgical specialty, and organ treated.
- Population characteristics, such as the number of patients and surgeries.
- Intervention characteristics, such as the type of minimally invasive surgical approach (laparoscopy or robot-assisted laparoscopy).
- Technology characteristics such as the type of AR navigation system used, the imaging guidance used (MRI, ultrasound, CT), and the use of a head-mounted display (HMD).
- Outcome characteristics, such as outcome accuracy and assessment of the control group.
- Other outcomes were usability, surgical outcome, operative time, complications, reoperation, and any specific complications related to AR system use.
- A conclusion, summary, and an assessment of study limitations.

### 2.10. Data Synthesis and Risk of Bias Assessment

Due to heterogeneity and significant methodological differences across studies, particularly in study designs, procedures, and technologies, a descriptive narrative synthesis is employed to better understand the use of augmented reality navigation in minimally invasive abdominal and thoracic soft-tissue surgery. Statistical testing of heterogeneity is unnecessary given the evident methodological differences across studies. Meta-analysis is not applicable due to the insufficient number of homogeneous studies with comparable procedures.

### 2.11. Related Works

An updated PROSPERO search (January 2026) confirmed our project is the only ongoing one in this area. No PubMed systematic reviews between 2014 and 2026 address AR for navigation in minimally invasive abdominal and thoracic soft-tissue surgery. To further assess the literature, a PubMed search on 23 February 2026, using our predefined inclusion criteria, showed publications on augmented reality remain concentrated in liver [18,19] and thoracic surgery [20,21].

## 3. Results

Out of 1297 studies screened, only 28 (2%) met the inclusion criteria and were ultimately included in this systematic review (Figure 2).

Studies focusing on minimally invasive abdominal and thoracic soft-tissue surgery span different domains, with 28% (*n* = 8) conducted using robot-assisted laparoscopic techniques and 71% (*n* = 20) performed using conventional laparoscopy. An overview of the 28 included studies is shown in Table 2.

### 3.1. AR Use in 28 Studies of Minimally Invasive Abdominal and Thoracic Soft-Tissue Procedures

The AR was used on the following organs and procedures within minimally invasive abdominal and thoracic soft-tissue surgery is shown in Table 3.

### 3.2. The Use of AR on Highly Mobile Anatomical Structures

Among the 28 included studies, AR was applied to deformable and mobile anatomy using five major strategies, shown in Table 4.

### 3.3. Devices Used in the 28 Included Studies

While 26 studies (92%) reported using static monitors as AR displays, 2 studies (7%) reported employing head-mounted displays (HMDs) as holographic AR displays in lung and liver surgery.

Expanding on the use of AR technologies, indocyanine green (ICG) fluorescence imaging combined with AR navigation was employed in four studies (14%), primarily in liver surgery. In these studies, real-time ICG fluorescence visualization was integrated with AR overlays to improve tumor boundary identification, vascular mapping, and resection accuracy. Hardware, software, AR navigation, and analysis/tracking systems are shown in Table 5.

### 3.4. Accuracy Levels and Usability Outcomes of the AR

Workflow efficiency regarding intraoperative setup and registration times was described qualitatively, without mean ± SD or statistical analysis [5,24,27,32,33,34,35,36,41,42,43]. 

Data showed that the combination of 3D printing and augmented reality reduces operative time (OT) [5], whereas intraoperative navigation and AccuVein show no meaningful impact on procedure duration [34,41]. 

Cognitive load, measured through surgeon feedback on usability and orientation, was collected via questionnaires and Likert scales but reported narratively, without nu-merical distributions or structured data. There was no formal evaluation of overall usabil-ity, cognitive load, or safety, nor were there structured surgeon satisfaction scores, stand-ardized workload measures, or objective assessments of mental or cognitive effort [24,25].

Surgeon-reported outcomes were not formally measured; impressions were presented only narratively without validated questionnaires [36]. Moreover, there was no formal assessment of anatomical understanding, training quality, or educational utility [37]. There was only indirect addressing of training quality and educational utility from a technolog-ical perspective, without validated anatomical comprehension assessments [48].

In terms of accuracy, the use of 3D models was associated with improved surgical margins [32,33]. 

A summary of reported accuracy levels and usability outcomes of AR systems is shown in Table 6.

### 3.5. Challenges in the Development and Effective Use of AR

Several methodological limitations were identified in the included studies regarding the use of AR systems for minimally invasive abdominal and thoracic soft-tissue surgery. Specifically, common issues included retrospective study designs, single-center settings, and non-randomized methodologies [5,6,7,26,38,41,42,43,44]. In addition, small sample sizes and the absence of control groups were noted in many studies [6,26,27,31]. Furthermore, surgery was often performed by a single surgeon or team [23,32,34,35,36,37,38,41,43]. Variable imaging and operative protocols, along with short follow-up durations, were also reported, limiting the assessment of long-term outcomes [40].

Technical limitations, including inadequate deformation tracking, rigid 3D models [37], experimental settings [22,29,33], and the need for manual adjustments [33,39] or additional personnel, may reduce workflow efficiency. Accuracy levels and usability outcomes are presented in Table 7.

## 4. Discussion

Substantial heterogeneity in the AR devices employed worldwide in minimally invasive abdominal and thoracic soft-tissue surgery between 2014 and 2024 was found. No publications meeting the inclusion criteria were identified within the field of gynecological surgery. However, a few small case series (*n* = 3) were published in 2017 using AR in gynecological surgery [7] and demonstrating AR-guided localization of small- and medium-sized intramural myomas during conventional laparoscopy. The software ran in real time on a standard Intel i7 PC and required no artificial landmarks. The preoperative uterus model was matched to the 3D model obtained during surgery using a semiautomatic registration process. This method required a small amount of manual input, in which the edges of the uterus were marked to help the system define the organ’s boundaries. The matched models were overlaid on each video frame, making the uterus appear partly transparent. This allowed the surgeon to clearly see the exact position of the myoma inside the uterus. This new AR system was able to track a highly mobile organ such as the uterus, something that had not been previously described.

Building on early gynecological AR research, later work introduced a robot-assisted AR system for identifying sentinel nodes during laparoscopic surgery. This 2022 study, using an animal model, used real-time multimodal fusion of laparoscopic images with preoperative imaging during pelvic lymphadenectomy. Notably, CT overlay accuracy was greater than 90%, and overlap rates were less than 6%. AR significantly improved the identification of target structures for both experienced surgeons and trainees [14].

However, published data within gynecological surgery remain preliminary and experimental, with small study cohorts limiting the level of evidence.

In addition to studies in gynecology, research using AR systems for minimally invasive soft-tissue surgery within the pelvic region has been conducted in urology [32,33,39]. For example, AR enhances the accuracy of targeted prostate biopsies by providing a 3D virtual reconstruction [33]. In robot-assisted radical prostatectomy (RARP), 3D model overlays, manually aligned with the endoscopic view, helped tailor nerve-sparing surgery and reduce positive surgical margins. This was especially useful for patients with extracapsular tumor extension [32,39]. The AR model correctly identified 70% of cancerous areas (sensitivity) and all healthy areas (100% specificity). Overall, it matched the actual cancer distribution 92% of the time (accuracy) [39]. These findings suggest AR systems are highly accurate and minimize false positives. This supports their use as a reliable surgical guidance tool.

Beyond pelvic surgery, nearly half of the studies in our review (*n* = 12, 42%) focused on AR in minimally invasive liver surgery. The anterior approach is often used to expose the inferior vena cava (IVC) and enable safer transection of the short hepatic veins. This includes en bloc caudate lobe resection. AR systems project the left-right liver demarcation line and the course of the middle hepatic vein (MHV) from the 3D model onto the liver surface, guiding the parenchymal transection plane. Despite inaccuracies caused by respiratory motion and tissue deformation, combining AR with intraoperative ultrasound (IOUS) enables effective guidance [6]. AR has also been linked to a lower incidence of bile leakage, likely due to improved protection of vascular structures and bile ducts [6,40].

As mentioned earlier, the implementation of AR in minimally invasive abdominal and thoracic soft-tissue surgery relies on several key technologies. Computational segmentation and 3D model creation, using methods such as SmartLiver surface-based registration, CNN-based segmentation, and 3D deformation modeling, allow precise identification of anatomical structures and support detailed 3D surgical planning [22,23,24]. Registration and tracking, employing AI-based segmentation and algorithms like weighted point-based registration and 3D positional measurements (e.g., CNN/iKidney, 2D U-net), align preoperative models with the patient’s anatomy. That provides accurate intraoperative guidance and improves safety [23]. Real-time motion compensation and continuous tracking update AR overlays during organ or camera movement. This helps maintain accurate anatomical guidance throughout the procedure [25,26]. Finally, real-time deformation tracking monitors tissue shifts using IOUS, intraoperative CT, fluorescence/ICG imaging, and endoscopic vision tracking. This ensures that anatomical models remain up to date, thereby improving surgical precision and safety [26,27,28,29,30,31].

When comparing AR platforms, this review found that the software and hardware used in AR-assisted surgery vary in several capabilities, including image processing, visualization, and the intended purpose of the AR system. Through segmentation, surgical planning, and intraoperative navigation, clinically validated software platforms such as Mimics and iPilot improve the accuracy and safety of surgery [5,28,30]. Moreover, MI-3DVS and simple DICOM viewers support quick anatomical review, saving time in the operating room [6,43]. Tools like vMIX enable real-time integration of AR [39]. Hardware platforms such as Fluid™ and AccuVein portable devices provide real-time feedback and improve visualization, with AccuVein specifically focusing on vascular visualization. Precision tracking systems such as Volga-M, Polaris Spectra, NDI Polaris Vicra, and Optotrak ensure real-time, accurate tracking of anatomy, supporting safe navigation. 3D printing platforms like OBJET500 allow creation of patient-specific models for preoperative planning [5,6,29,37,45]. Intraoperative imaging systems DynaCT and PINPOINT/DPM-III-01 provide high-resolution updates and real-time perfusion assessment, which are important for surgeons to adapt to changes during surgery. Together, these tools improve anatomical mapping and guide interventions, thus reducing risks in complex surgical procedures.

Despite the advances described above, each AR platform also has limitations. AR-assisted navigation systems (AR-ANS) developed by individual research teams may be difficult to generalize and often lack regulatory approval. Generating accurate 3D models requires close collaboration between radiologists, surgeons, and engineers. Currently, superimposition of 3D virtual models (3DVMs) onto the operative field is often performed manually. This process typically requires assistance. Clinical platforms can be costly, less flexible, and tied to specific hardware. DICOM-based systems used in urological surgery can be complex without proper software support [23]. Intraoperative reliability and precision could be improved through fully automatic model superimposition [24,32].

Taken together, the evidence presented shows that integrating deformable anatomical tracking with multimodal intraoperative data fusion has significant practical implications for safe and effective AR in surgery. These systems maintain accurate alignment, even in the presence of organ motion, deformation, or surgical manipulation, and provide reliable visual guidance. Real-time updates from video, fluorescence, ultrasound, or CT keep critical structures visible as anatomy changes. This reduces the risk of injury and improves precision. AI-enhanced platforms—such as Therapixel™, Visible Patient™, IGNITE, iKidney, and SmartLiver—create accurate 3D models from multimodal data. They support manual, semi-automatic, or fully automatic registration using internal landmarks, vessel positions, and surface points [23,24]. These features enhance the consistency, usability, and reliability of AR systems. When combined with robotic surgery, which stabilizes the endoscopic camera, AR overlays remain consistent, further improving surgical planning, precision, and safety.

### 4.1. Training and Education

This review provides only a narrative description of educational utility, without any validated objective performance metrics that would allow for a quantifiable assessment of trainee skills (e.g., precision, task completion, errors) or support consistent monitoring of learning progression. The study by Iop et al. [48] demonstrates that XR simulation can complement traditional training by providing evidence-based, data-driven feedback that facilitates skill acquisition in a safe, controlled environment, without risk to patients. The educational benefit lies in the use of objective performance metrics rather than in the formal assessment of cognitive or theoretical learning outcomes.

### 4.2. Cost–Benefit Analysis

None of the included studies presented cost–benefit calculations. The 2025 paper [49] presents a study protocol detailing planned cost–benefit and cost-effectiveness analyses comparing AR surgical navigation, conventional navigation, and the free-hand technique using QALYs and ICER metrics. As this is a protocol publication and not a report of trial results, the actual cost–benefit outcomes are still pending and will be available only after data collection and analysis are complete. According to the protocol, cost data will be collected at discharge and at 30, 90, and 365 days. These include inpatient and outpatient care costs, medications, sick leave, lost productivity, and total patient costs. Quality-adjusted life years (QALYs) will be calculated with the EQ-5D-3L instrument and, together with cost data, used to estimate the incremental cost-effectiveness ratio (ICER) between surgical techniques.

## 5. Limitations

Included studies on AR systems for minimally invasive abdominal and thoracic soft-tissue surgery showed methodological weaknesses. Most were retrospective, single-center, non-randomized, with small samples and limited follow-up, reducing statistical power and generalizability. Many lacked control groups. Procedures were often performed by a single surgeon or team, potentially introducing performance bias. Technical inconsistencies harmed accuracy, especially with variable imaging and rigid 3D models. Most platforms were experimental, needed frequent manual recalibration, and relied on extra staff and specialized hybrid theatres. High development and equipment costs further limited AR use in major academic centers.

## 6. Conclusions

Advancing AR requires larger, rigorously designed clinical studies that use standardized protocols, focus on relevant clinical outcomes, and include multicenter collaboration to strengthen the evidence base and support reliable adoption in gynecological surgery.

Evidence from urological procedures also demonstrates that AR enhances visualization and precision in minimally invasive pelvic surgery. Reports of high specificity and overall accuracy suggest that AR can serve as a reliable adjunct for intraoperative guidance, though broader validation is still needed to confirm its clinical impact.

In thoracic surgery, the combination of AR, 3D printing, and multimodal imaging has been shown to enhance anatomical visualization and support intraoperative localization of challenging lung nodules, suggesting the potential to improve surgical outcomes.

In minimally invasive liver surgery, AR is utilized to define anatomical resection planes and protect critical vascular structures. Despite persistent technical challenges, particularly those arising from organ motion and deformation, integrating AR with intraoperative ultrasound appears to improve procedural accuracy.

In summary, integrating deformable anatomical tracking with multimodal intraoperative data and 3D modeling enhances the clinical potential of AR in precision surgery. However, technical variability, frequent recalibration requirements, high costs, and dependence on specialized personnel limit its use. That keeps most AR systems experimental and confined to major centers. Future AR platforms should be affordable and compatible with standard operating rooms to ensure access for everybody to all healthcare systems.

## 7. Future Perspective

Future studies should prioritize prospective, multicenter randomized trials with sufficient sample sizes and extended follow-up. When possible, incorporate suitable control groups and blinded outcome assessments. Procedures should involve multiple surgeons with standardized training and be evaluated using objective, uniform metrics to enable comparison and minimize performance bias [48]. To date, evidence in gynecologic and urological surgery remains limited; however, emerging studies indicate these fields are beginning to evaluate and implement such techniques. These represent key areas for future investigation within minimally invasive soft-tissue pelvic surgery.

Technical robustness can be improved through developing deformable, real-time models and standardized imaging and registration protocols. Automated tracking methods may reduce dependence on manual recalibration. Ongoing research should aim to design AR platforms that operate reliably in standard operating rooms without specialized hybrid theatres or extra personnel. Cost-effective, interoperable hardware and software will support broader clinical use. Finally, transparent reporting of AR system performance and limitations is crucial to enable integration into routine surgical practice.

## Figures and Tables

**Table 1:** Inclusion and exclusion criteria according to the PICO (population, intervention, comparators, outcome) model.

Criteria	Inclusion	Exclusion
**Study type**	Published peer-reviewed and original studies, randomized as well as observational studies, including case series *n* ≥ 4.	Non-original publications such as reviews, editorials and letters to the editor along with conference abstracts, unpublished studies as well as technical reports and earlier systematic reviews. Studies on the use of AR in orthopedic surgery, neurosurgery, vascular surgery, and plastic surgery were excluded, as were studies conducted on animals, cadavers, or phantoms. Case reports and series < 4.
**Year of publishing**	2014–2024	Before 2014
**Language**	English	All other languages
**Device**	Hardware and software within the augmented reality	Not applicable
**Population**	Aged 18 to 64 years	Aged 17 years or younger Aged 65 years or older
**Intervention**	Use of AR on humans within minimally invasive abdominal and thoracic soft-tissue surgery	Use of virtual reality (VR), mixed reality (MR)
**Comparator**	Comparison between groups with and without AR, types of devices used	Not applicable
**Outcome**	Knowledge generation in minimal invasive precision surgery (accuracy, usability)	Not applicable

**Table 2:** Overview of the 28 included studies. Baseline characteristics.

Author	Country	Number of Patients	Organ	Kind of Surgery	Study Intention	Kind of Surgery	Navigation System	Head mounted-Display (HMD)
Li 2021 [5]	China	*n* = 142	Lungs	Thoracic Surgery	Visualizing the patients’ interior lung structure on the TV screen versus 3D-printed models using AR platform	Thoracoscopic surgery	MIMICS17.0 software, OBJET500 3D printer, Medical-Professional Augment Reality Display & Interaction Device	yes
Yasuda 2020 [6]	Japan	*n* = 8	Liver	Liver Surgery	Image-guided navigation system (IG-NS)	Laparoscopic liver surgery	Digital Imaging and Communications in Medicine (DICOM). Image segmentation by Analyze. Image-guided navigation system (IG-NS). 3D positional measurement by Optotrak.	yes
Amparore 2024 [22]	Italy	*n* = 20	Kidney	Urologic Surgery	Automatic integration of 3D virtual models into the Da Vinci robotic console	Robot-assisted kidney surgery	Computer vision technology and convolutional neural network technology	no
Piana 2024 [23]	Italy	*n* = 13	Kidney	Urologic Surgery	Convolutional neural networks (CNNs) in automatic augmented-reality 3D virtual models	Robot-assisted partial nephrectomy	Hyper-accuracy 3D models (HA3D™). Three-dimensional virtual models (3DVMs) were processed in DICOM format, convolutional-neural-network (CNN) and iKidney software	no
Schneider 2020 [24]	United Kingdom	*n* = 18	Liver	Liver Surgery	Manual and semi-automatic registration	Laparoscopic liver surgery	SmartLiver	no
Baste 2018 [25]	France	*n* = 4	Lungs	Thoracic Surgery	Three-dimensional (3D) imaging throughout the surgical procedure	Robot-assisted thoracic surgery (RATS), segmentectomies	Hardware platform FluidTM	no
Deng 2024 [26]	China	*n* = 16	Liver	Liver Surgery	ARN and ICG fluorescence imaging	Laparoscopic hepatobiliary surgery	Medical Image 3D Visualization System (MI-3DVS) software, developed by authors	no
Bijlstra 2022 [27]	The Netherlands	*n* = 15	Liver	Liver Surgery	Robotic Liver Surgery Cockpit	Robot-assisted liver surgery	MeVisLab framework software, /ParaView Glance. Tilepro, deep learning approach using a 2D U-net	no
Chen 2014 [28]	China	*n* = 15	Kidney	Urologic Surgery	Three-dimensional model of renal tumor facilitating surgical planning and imaging guidance	Laparoscopic kidney surgery	Mimics 12.1 software	no
Prevost 2019 [29]	Switzerland	*n* = 9	Liver	Liver Surgery	Intraoperative 3D imaging i combination with IGS	Laparoscopic liver surgery	CAS-One AR system, NDI Polaris Vicra optical tracking system	no
Rouzè 2016 [30]	France	*n* = 8	Lungs	Thoracic Surgery	Intraoperative cone beam computed tomography (CBCT) and augmented fluoroscopy	Video-assisted thoracic surgery (VATS)	DynaCT acquisition system, iPilot segmentation software	no
Tao 2023 [31]	China	*n* = 31	Liver	Liver Surgery	AR-assisted navigation system combined with indocyanine green fluorescence imaging (FI)	Laparoscopic liver surgery	ARN–FI	no
Checcucci 2022 [32]	Italy	*n* = 160	Prostate	Urologic Surgery	Role of 3D models on positive surgical margin rate (PSM)	Robot-assisted prostate surgery	High-definition Hyper-Accuracy 3D model (HA3D^®^) ™	no
Porpiglia 2018 [33]	Italy	*n* = 30	Prostate	Urologic Surgery	Hyper-accuracy three-dimensional (HA3D) reconstruction based on multiparametric magnetic resonance imaging (mpMRI) and superimposed imaging	Robot-assisted prostate surgery	pViewer application software, developed using the Unity platform and C Sharp	no
Huber 2023 [34]	Germany	*n* = 16	Liver	Liver Surgery	The quotient of intraoperative resected volume and planned resection volume	Laparoscopic liver resection	CAS-One Liver	no
Lin 2018 [35]	Taiwan	*n* = 19	Suprarenal (adrenal) gland	Urologic Surgery	AR-assisted navigation system	Laparoscopic adrenalectomy (AR-SILA)	3D virtual patient modeling (VPM), AR software	no
Zhang 2021 [36]	China	*n* = 85	Liver	Liver Surgery	Laparoscopic augmented reality navigation (LARN) system	Laparoscopic liver surgery	The ARN system, developed by authors	no
Dubrovin 2019 [37]	Russian Federation	*n* = 12	Kidney	Urologic Surgery	Original hardware–software complex	Laparoscopic partial nephrectomy	Hardware–software complex ‘Volga-M’	no
Wang 2024 [38]	China	*n* = 98	Liver	Liver Surgery	AR-assisted navigation system combined with indocyanine green fluorescence imaging (FI)	Laparoscopic liver surgery	3D visualization system, PINPOINT imaging system or DPM-III-01 imaging system	no
Schiavina 2021 [39]	Italy	*n* = 26	Prostate	Urologic Surgery	3D model with AR (AR-3D model) to guide nerve sparing (NS)	Robot-assisted prostate surgery	vMIX software	no
Wu 2023 [40]	China	*n* = 82	Pancreas	Pancreas Surgery	AR-assisted navigation system	Laparoscopic pancreatoduodenectomy	The AR-ARS system, developed by authors	no
Law 2018 [41]	Canada	*n* = 724	Abdominal wall hematoma	Urologic Surgery	Pre-incision imaging with AccuVein compared to transabdominal illumination	Abdominal wall hematoma at robot-assisted radical prostatectomi	AccuVein AV400	no
Wu 2022 [42]	China	*n* = 77	Liver	Liver Surgery	AR-ANS (augmented reality–assisted navigation system)	Laparoscopic liver surgery	The AR-ARS system, developed by authors	no
Wang 2023 [43]	China	*n* = 11	Liver	Liver Surgery	AR-assisted navigation system	Laparoscopic liver surgery and total caudate lobectomy	MI-3DVS software and an ARN system, developed by authors	no
Tao 2021 [44]	China	*n* = 43	Spleen	Pancreatic Surgery	AR-assisted navigation system	Laparoscopic spleen surgery	The ARLN system	no
Hayashi 2016 [45]	Japan	*n* = 20	Ventricle	Gastric Surgery	Registration accuracy in the surgical navigation	Laparoscopic ventricle surgery	Weighted point-based registration method for computing the transformation matrix using fiducials on body surface and blood vessels	no
Zhu 2023 [46]	China	*n* = 76	Liver	Liver Surgery	AR-assisted navigation system combined with indocyanine green fluorescence imaging (FI)	Laparoscopic liver surgery	The ARN system, developed by authors	no
Hayashi 2016 [47]	Japan	*n* = 23	Ventricle	Gastric Surgery	Surgical navigation system synchronized with the laparoscope position	Laparoscopic ventricle surgery	Polaris Spectra optical tracking system, NDI 6D Architect software	no

**Table 3:** Use of Augmented Reality in 28 Studies of Minimally Invasive Abdominal and Thoracic Soft-Tissue Procedures, by Organ and Procedure.

Surgical Site	Organ (Number of Studies)	Organ Involved
**Upper abdomen**	Liver (12)	Liver resection
	Ventricle (2)	Ventricle surgery
	Pancreas (1)	Pancreatoduodenectomy
	Spleen (1)	Spleen surgery
**Pelvis/Retroperitoneum**	Kidney (4)	Partial nephrectomy
	Prostate (3)	Prostate surgery
	Adrenal gland (1)	Adrenalectomy
**Thorax**	Lung (3)	Thoracic surgery
**Other**	Abdominal wall hematoma (1)	Urologic surgery

**Table 4:** Methods for managing soft-tissue deformation and maintaining AR accuracy during minimally invasive abdominal and thoracic surgery on highly mobile anatomical structures.

Strategy	Description of the Strategy	Examples/References
**Computational Segmentation or 3D Model Creation**	Predictive models estimate tissue deformation using surface registration, AI segmentation, and 3D simulations	SmartLiver surface-based registration, CNN-based segmentation, 3D deformation modeling [22,23,24]
**Real-Time Motion Compensation and Continuous Updating**	AR overlays are continuously recalibrated using computer vision, laparoscope tracking, and real-time fusion with preoperative 3D models	Real-time alignment via computer vision, continuous tracking, algorithmic overlay fusion [25,26]
**Real-time Deformation Tracking**	Deformation is tracked using multiple intraoperative imaging modalities providing real-time anatomical updates	IOUS, intraoperative CT, fluorescence/ICG imaging, real-time endoscopic vision tracking [26,27,28,29,30,31]
**Surgeon-Guided Recalibration**	Manual recalibration when automated tracking fails, including realignment, fusion, re-registration, and marking	Manual realignment in AR-RARP, manual fusion on secondary screen, tape-based anatomical marking [24,32,33]

**Table 5:** Overview of hardware, software, AR/navigation, and analysis/tracking systems identified in the included studies.

Category	Systems/Platforms	Characteristics
**Hardware Platforms**	Fluid™, Volga-M, Polaris Spectra, AccuVein, OBJET500, NDI Polaris Vicra, DynaCT, PINPOINT/DPM-III-01, Optotrak	Optical trackers, imaging systems, and illumination devices for AR integration
**Software Platforms/Frameworks**	MeVisLab, ParaView, Mimics, MI-3DVS, iPilot, vMIX, pViewer, DICOM	Image processing, segmentation, visualization, and integration with AR workflows
**AR/Navigation Systems**	HA3D^®^, VPM, CAS-One Liver/AR, SmartLiver, ARLN, ARN–FI, AR-ARS, ARN, IG-NS	Navigation and guidance systems providing intraoperative AR overlays
**Analysis/Registration/Tracking/AI Systems**	CNN/iKidney, 2D U-net, weighted point-based registration, 3D positional measurement	AI-based segmentation, registration algorithms, and tracking methods
**AR-ARS Systems Developed by Study Authors**	Six devices developed by respective research teams	Custom AR-ARS systems purpose-built within the included studies

**Table 6:** Summary of reported accuracy levels and usability outcomes of AR systems in minimally invasive abdominal and thoracic soft-tissue surgery.

Domain	AR Key Findings	Examples/References	Mean ± SD
**Workflow** **Efficiency,** **Operative** **Times**	Do not extend operative time (OT) and occasionally reduced it	Reduction in OT i 3D printing and AR group [5] Reduced time reported in AR-SILA procedures [35] No significant difference in navigation group [36,41]	3D printing and AR group (65.00–87.25 min vs. 78.00–107.25 min; *p* = 0.001) [5]. 150 min (range 75–330 min) [24] 192 min (135–263) [27] Navigated group 189 min (103–267); non-navigated group: 180 min (103–288), *p* = 0.970 [34]. Single-incision laparoscopic adrenalectomy (AR-SILA) group (102.5 min ± 12.8) vs. nonAR-SILA 150.9 min ± 46.3 (*p* = 0.005) [35]. The intraoperative navigation (IN) group 300 min (90–690); non-navigated (NIN) group 300 min (90–540), *p* = 0.061 [36]. Controll group (*n* = 610) = 162 min; AccuVein group (*n* = 114) = 159 min (*p* = 0.54) [41].
**Cognitive Load**	Decrease cognitive load and improve intuitive navigation	Reduction in hand–eye discordance observed with ARLN and LARN [36]	Surgeons can keep track of the surgical field without the distraction during surgical procedure, solve the hand-eye incongruity during laparoscopic surgery [36].
**Surgeon-Reported Outcomes**	High surgeon satisfaction	Reported improvements in technical accuracy, intraoperative time efficiency, and situational awareness [5,24]	Higher evaluation scores in the 3D printing and AR group (96.00–98.00 vs. 89.75–92.00; *p* = 0.001) [5]. Qualitative surgeon feedback; no statistical summaries [24].
**Cross-Modality Reliability**	Reliable across surgical modalities	Effective in laparoscopic and robotic procedures [24,32,33,42]	Registration accuracy (manual vs. semi-automatic) Manual Target Registration Error phase1 15.8 ± 7.2, phase2 10.9 ± 4.2 [24]. Surgical margin outcomes and changes in surgical technique choices (*p* = 0.01 for lower positive surgical margins in the 3D group) [32]. Spatial concordance between the 3D model and actual specimen (e.g., mismatch < 3 mm over 85% of surface) [33]. An intuitive navigation interface, can adjust the preoperative 3D model’s size, transparency, rotation, and translation [42].
**Educational Utility**	Improved anatomical understanding and training quality	Benefits observed with AR and 3D-printed anatomical models [36,37,48]	Narrative description only [36,37,48].
**Overall Assessment**	Usable, safe, and beneficial without adding cognitive or time burden	Offered improved visualization and workflow optimization [5,24,43]	Less blood loss in the 3D printing and AR group (22.00–46.25 mL vs. 33.75–60.00 mL; *p* = 0.006), shorter length of hospital stay (3–4 days vs. 4–5 days; *p* = 0.001) [5]. Mean registration accuracy 10.9 ± 4.2 mm (manual) vs. 13.9 ± 4.4 mm (semi-automatic). Mean difference 3 mm; *p* = 0.158 [24]. Negative surgical margins, no conversion to open surgery. Lower intraoperative blood loss in the ARN-FI group (100 vs. 200 mL, *p* = 0.005), lower incidence of remnant liver ischemia (13.3% vs. 30.2%, *p* = 0.046) [43].

**Table 7:** Overview of the 28 included studies. Accuracy levels and usability outcomes.

Study ID	Outcome	Usability	Limitations
Li C 2021 [5]	Combining AR and 3D printing enhanced surgeons’ understanding of lung anatomy, potentially improving surgical outcomes.	3D printing and AR support medical education and simulation, providing students with tangible lung models to study internal structures.	Retrospective study. Singel center. Small arteries, veins, and bronchi may not be replicated as in the computer or AR models. All surgeries were performed by five surgeons
Yasuda 2020 [6]	Developed tape method using surgical cloth to mark distances along anatomical features. IG-NS required ≥3 registration points, with repeated registration during surgery to reduce error.	Repeated registration using non-liver surface points improved navigation accuracy during hepatectomy.	ּA single-center retrospective cohort study. ּSmall number of patients.
Amparore 2024 [22]	CNN proved superior to computer vision in identifying target organ boundaries without the need for indocyanine injection.	CNN detects the target organ using intrinsic pixel characteristics instead of ICG-dependent visualization. Enables fully automatic AR procedures, as each organ pixel can be independently identified without specific landmark.	Predominance of red tones in endoscopic images impairs segmentation. Light variation, endoscope motion, and ICG-related color differences affect accuracy. Requires a stationary, unobstructed target organ. Software remains at an experimental stage.
Piana 2024 [23]	Ikidney, a CNN-based software, was created to generate highly accurate 3D models for automatic AR-guided RAPN. Indocyanine green fluorescence (ICG) was used for kidney identification and the Ikidney software development.	The CNN-based software identifies every kidney pixel without relying on landmarks. Organ position, rotation were manually taken from still frames from the RAPN video bank, and used to train the neural network. The automatic ICG-guided AR system anchored the 3DVM to the real kidney without human input.	All surgery was performed by a single surgeon. An assistant was needed to fine-tune the 3DVM overlay. ICG use may accentuate microvascular variations, causing overlap errors. High development costs limit the reproducibility of this technology outside academic centers.
Schneider 2020 [24]	SmartLiver supports manual and semi-automatic registration. Semi-automatic uses 3D laparoscope images to create a liver surface point cloud, aligned with the preoperative model via the ICP algorithm.	Semi-automatic registration could improve SmartLiver usability.	Accuracy is comparable to manual registration, but larger studies are needed for confirmation. Not tested for robotic-assisted surgery.
Baste 2018 [25]	This multimodal imaging protocol simplifies the complex workflow by integrating Therapixel™ and Visible Patient™.	Combining a multimodal imaging system with robotic surgery may enhance segmentectomy performance.	Descriptive user study; high-cost limits technology development.
Deng 2024 [26]	3D visualization and augmented reality navigation help to understand complex anatomy and plan appropriate surgical approaches for IHD stones.	Preoperative 3D planning and ARN-guided dissection preserved caudate bile ducts, ensured safe LHD closure, and prevented bile duct injury or stricture. ARN combined with ICG imaging improves the safety and precision of LLH for hepatolithiasis.	Mean reprojection accuracy of 10 mm. The small sample size limits evaluation of long- and short-term outcomes.
Bijlstra 2022 [27]	Interactive VR system for 3D reconstruction of preoperative CT, MR, and PET-CT imaging, enabling surgeons to view the operation field, 3D images, fluorescence imaging, and IOUS simultaneously.	In-house segmentation and 3D modeling of preoperative CT and MRI are feasible, and the da Vinci robot effectively integrates VR 3D models, IOUS, and ICG-fluorescence imaging.	Small sample size and no control group. Variable imaging protocols. Lack of real-time visualization.
Chen 2014 [28]	Real-time manual image fusion on a separate screen did not burden the surgeon or delay the procedure.	Superimposing 3D images on the operative view enhances anatomical localization and surgical accuracy through augmented reality image fusion.	The method is labor intensive and lacks a control group.
Prevost 2020 [29]	Landmark-based AR in 3D laparoscopic liver surgery is feasible with minimal workflow impact and offers substantial benefit for detecting vanishing lesions.	Intraoperative time efficiency. Technical accuracy. Clinical benefit and surgeon satisfaction.	Not yet sufficiently advanced to guide complex liver resections. Absence of dynamic, non-rigid registration, overlay inaccuracies.
Rouzè 2016 [30]	Pulmonary nodule identification on the intraoperative CBCT.	Resection of hard-to-palpate lung nodules improves when paired with AR. This early experience shows CBCT’s potential to enhance tumor localization during VATS.	Feasible in hybrid operating theatres only, which are not widely used in routine thoracic surgery.
Tao 2023 [31]	Intraoperative blood loss was significantly lower in the ARN–FI group, while other intraoperative variables (resection type, transfusion, and conversion to open surgery) did not differ between groups.	Although ARN with ICG fluorescence did not reduce operative time, it decreased intraoperative bleeding and improved resection accuracy, effectively guiding laparoscopic segment 8 resection.	ARN reprojection error was <10 mm. It may still affect fine surgical precision and require IOUS support. The small sample size and single-center design limit statistical power and generalizability.
Checcucci 2022 [32]	AR-RARP was achieved by real-time intraoperative overlay of 3D models within the robotic console, manually aligned with endoscopic images using a 3D professional mouse.	Intraoperative 3D models help tailor the nerve-sparing approach and reduce positive surgical margins, particularly in patients with extracapsular tumor extension.	All patients in control group were treated by the same surgeon.
Porpiglia 2018 [33]	Accuracy of index-lesion localization. Confirmation of suspected extracapsular extension (ECE). Accuracy of AR-guided selective biopsies at the neurovascular bundles. Spatial accuracy of the 3D virtual reconstruction for AR guidance.	AR can be used during surgery to support the learning curve and improve functional and oncological outcomes.	Repeat high-resolution mpMRI for HA3D reconstruction requires. Tumor segmentation is manually performed, operator dependent. The 3D model needs constant intraoperative adjustment by an assistant. No automated, elastic model exists to account for organ motion. Lack of integration with the robotic console limits full intraoperative use.
Huber 2023 [34]	Intraoperative 3D navigation did not affect the surgical plan.	Technology is safe for open and laparoscopic cases.	Single center randomized pilot trial with limited value in a controlled setting of two expert HPB surgeons.
Lin 2018 [35]	AR-SILA gives surgeons intraoperative guidance to locate lesions and vessels beyond direct vision.	Operative time was reduced with AR-SILA compared to SILA.	Surgeries were performed by a single surgeon.
Zhang 2021 [36]	In the IN group, LARN fused 3D models with the surgical field in real time, visualizing lesion–vessel relationships and predicting critical vessels to prevent bleeding and hepatic ischemia.	LARN allows surgeons to monitor the surgical field continuously, reducing hand-eye incongruity during critical laparoscopic steps.	Retrospective case–control study. All surgeries were done by a single laparoscopic team. LARN is limited by slight lag and imperfect alignment with liver shifts from pneumoperitoneum, respiration, heartbeat, and manipulation.
Dubrovin 2019 [37]	A 3D organ model aids surgical planning and patient education.	A 3D organ model supports surgical planning and patient education. Our hardware–software system enables effective visualization during organ-preserving urologic surgery.	Augmented reality use in surgery is challenged by real-time integration of 3D models with live images. This single-center, prospective, nonrandomized study lacked a control group.
Wang 2024 [38]	Functional liver parenchyma was maximally preserved while ensuring a negative margin and complete removal of the segment from peripheral to primary Glissonean branches.	The ARN system integrates the 3D model with the laparoscopic view and ICG FI in real time, projecting vessels, tumors, and liver segments onto the liver surface and indicating dissection depth, enabling precise execution of preoperative plans.	Single-center retrospective cohort study. Data analyzed retrospectively. All surgeries performed by one surgeon.
Schiavina 2021 [39]	The concordance between the 3D model and the whole-mount specimen showed 70% sensitivity, 100% specificity, and 92% accuracy in the prostate map analysis.	AR-3D–guided surgery improves real-time index lesion identification and can alter the NS approach.	Small patient cohort. No control group. Use of rigid 3D prostate models that ignore tissue deformation and lack surgical realism. Manual adjustment affects navigation precision.
Wu 2023 [40]	Lower blood loss in the AR group may contribute to group differences. AR-ANS helps locate and assess the pancreatic duct, enabling drainage tube placement and protecting the duct without direct anastomosis.	The surgeon can assess the position, quantity, and diameter of the pancreatic duct during operation, which is advantageous for the protection of the pancreatic duct. The AI-powered AR navigation system enhances image integration, reduces manual correction time, and improves intraoperative navigation efficiency.	Retrospective cohort study. Short follow-up.
Law 2018 [41]	Imaged with the AccuVein system pre-incision, only 2.6% developed AWH.	The AccuVein infrared system is limited to visualizing vasculature up to 10 mm beneath the skin.	Retrospective, non-randomized study Surgeries performed by a single surgeon. Anticoagulant therapy was discontinued preoperatively.
Wu 2022 [42]	AR-ANS integrated the 3D model with the intraoperative view to locate bile ducts, stones, and the resection plane, while ultrasound identified vessel orientation, helping accurately translate preoperative planning into surgery.	AR improved surgical safety by reducing blood loss and need for blood transfusions. The 3D model can be overlaid on laparoscopic and fluorescence images, enabling AR-guided liver resection planning and bile leakage detection.	A single-center retrospective cohort study.
Wang 2023 [43]	The liver contour, vena cava fossae, and gallbladder were used for registration, with manual adjustment if automatic results were unsatisfactory. The fusion algorithm then updated the spatial alignment in real time to track laparoscopic motion.	Combining ARN with IOUS improves the accuracy of the parenchymal transection plane during hepatectomy.	Two senior surgeons performed all operations. The approach appears feasible for selected patients but may not suit pCCA cases requiring vascular reconstruction. The small sample size limits power analysis.
Tao 2021 [44]	ARLN improves image–action coordination and adapts visualization without requiring constant shifts in the surgeon’s attention.	MI-3DVS can measure the splenic pedicle width to help decide if it should be ligated before using the Endo-GIA. The ARLN system provides a single-screen display, reducing hand–eye coordination demands and enabling the entire team to view merged images and the surgical field simultaneously, thereby enhancing collaboration and minimizing distraction.	Retrospective single-center study. Small number of patients.
Hayashi 2016 [45]	Vessel positions along with skin-surface landmarks, with vessel locations identified after surgical cutting.	Utilizing blood vessel positions reduces registration error and enhances navigation accuracy.	Only cut vessel positions were used as internal landmarks. Internal anatomical fiducials are not used at the beginning of surgery. Procedures were performed by the same surgeon.
Zhu 2023 [46]	Fluorescence imaging defined tumor boundaries for radical resection. In the ARN-FI group, it detected three small tumors missed by preoperative imaging, including one type I CL-HCC and a small S2 tumor.	3D visualization and AR accurately display major hepatic vein branches. For smaller veins, controlling central venous pressure helps reduce vein diameter, bleeding, and air embolism risk.	Small sample size. Technical limitations include ARN registration inaccuracy and high fluorescence false-positive rate.
Hayashi 2016 [47]	Progressive internal landmark registration maintains accuracy even if landmarks are moved or removed. Using blood vessel positions reduces registration error and improves navigation.	Progressive registration may apply to various endoscopic procedures. This study used only cut vessel positions, though visible organ surface features could also serve as internal landmarks.	A limitation of this protocol is the initial lack of internal fiducials. Landmark identification by the operating surgeon ensured stability.

## Supplementary Materials

The following supporting information can be downloaded at: https://www.mdpi.com/article/10.3390/s26061962/s1, This review was conducted in accordance with the PRISMA 2020 guidelines. The completed PRISMA checklist and additional methodological details are provided as Supporting Information files: File S1: PRISMA 2020 Checklist; File S2: Detailed description of the search strategy development; File S3: Database search queries; File S4: Inclusion and exclusion criteria.
