# Supplementary material for: The Use of Augmented Reality for Navigation in Minimally Invasive Abdominal and Thoracic Soft-Tissue Surgery: A Systematic Review
Source: Sensors (Basel). 2026 Mar 20;26(6):1962. doi: 10.3390/s26061962 (PMC13030279; doi:10.3390/s26061962)
Supplement: Supplementary file 1 [file sensors-26-01962-s001.zip › Supplementaly_file_S2_description_of_the_search_strategy.pdf]

Description of Search Strategy

In information retrieval, precision is defined as the proportion of retrieved records that are relevant, whereas recall is defined as the proportion of all relevant records that are successfully retrieved. Irrelevant records retrieved by a search are considered false positives and reduce precision, while relevant records that are not retrieved are considered false negatives and reduce recall.

Based on these definitions, three electronic bibliographic databases relevant to the medical domain -PubMed, Web of Science, and Embase -were systematically searched. An initial broad set of keywords and search fields was developed based on the research question and intended application domain. The search was limited to studies published between 2014 and 2024 and to articles written in English. All database searches were completed on October 28, 2024. The full electronic search strategies for all databases are provided in Supplementary file 3.

The database search yielded 2,134 records. After removal of duplicates, 1,297 records remained and were uploaded to the Rayyan web-based systematic review platform. Records were screened sequentially by title and abstract by two independent, non-blinded reviewers (I.S. and V.G.E.-H.) according to predefined inclusion and exclusion criteria based on the PICO (population, intervention, comparator, outcome) framework. Disagreements regarding study eligibility for two records were resolved through discussion and, when necessary, consultation with a third reviewer (K.A.R.-W.).

Following title and abstract screening, 28 articles were retained for full-text assessment. Full-text data extraction was conducted independently by two non-blinded reviewers (I.S. and L.C.). All 28 studies met the predefined inclusion criteria and were included in the final qualitative synthesis. The study selection process followed the PRISMA 2020 guidelines.
