# Supplementary material for: The Use of Augmented Reality for Navigation in Minimally Invasive Abdominal and Thoracic Soft-Tissue Surgery: A Systematic Review
Source: Sensors (Basel). 2026 Mar 20;26(6):1962. doi: 10.3390/s26061962 (PMC13030279; doi:10.3390/s26061962)
Supplement: Supplementary file 1 [file sensors-26-01962-s001.zip › Supplementary_file_S3_database_search_queries.pdf]

PubMed,<br>Web of Science,<br>Embase	"augmented reality"[MeSH Terms] OR "augmented reality" OR augmented-reality) AND ("GI" OR gastrointestinal OR digestive OR abdominal OR urological OR colorectal OR thoracic OR chest OR "gynecology"[MeSH Terms] OR gynaecolog* OR "neoplasms"[MeSH Terms] OR cancer OR oncolog* OR tumor) AND (laparoscop* OR robot laparoscop OR minim* invasive OR robot*)
