# Supplementary material for: The Use of Augmented Reality for Navigation in Minimally Invasive Abdominal and Thoracic Soft-Tissue Surgery: A Systematic Review
Source: Sensors (Basel). 2026 Mar 20;26(6):1962. doi: 10.3390/s26061962 (PMC13030279; doi:10.3390/s26061962)
Supplement: Supplementary file 1 [file sensors-26-01962-s001.zip › Supplementary_file_S4_inclusion_and_exclusion_criteria.pdf]

Inclusion and exclusion criteria according to the PICO (population, intervention, comparators, outcome) model.

Criteria	Inclusion	Exclusion
**Study type**	Published peer-reviewed and original studies, randomized as well as observational studies, including case series n ≥4.	Non-original publications such as reviews, editorials and letters to the editor along with conference abstracts, unpublished studies as well as technical reports and earlier systematic reviews. Studies on the use of AR in orthopedic surgery, neurosurgery, vascular surgery, and plastic surgery were excluded, as were studies conducted on animals, cadavers, or phantoms. Case reports and series <4.
**Year of publishing**	2014-2024	Before 2014
**Language**	English	All other languages
**Device**	Hardware and software within the augmented reality	Not applicable
**Population**	Aged 18 to 64 years	Aged 17 years or younger<br>Aged 65 years or older
**Intervention**	Use of AR on humans within minimally invasive abdominal and thoracic soft-tissue surgery	Use of virtual reality (VR), mixed reality (MR)
**Comparator**	Comparison between groups with and without AR, types of devices used	Not applicable
**Outcome**	Knowledge generation in minimal invasive precision surgery (accuracy, usability)	Not applicable
